# A Mediterranean-Style Diet Improves the Parameters for the Management and Prevention of Type 2 Diabetes Mellitus

**DOI:** 10.3390/medicina59101882

**Published:** 2023-10-23

**Authors:** Stefani A. Derrick, Sarah T. Nguyen, Jordan R. Marthens, Leah L. Dambacher, Angelos K. Sikalidis, Scott K. Reaves

**Affiliations:** 1 Department of Food Science and Nutrition, California Polytechnic State University, San Luis Obispo, CA 93407, USA; sderrick@calpoly.edu (S.A.D.);; 2 Center for Health Research, California Polytechnic State University, San Luis Obispo, CA 93407, USA; 3 Personalized Nutrition Research Group, California Polytechnic State University, San Luis Obispo, CA 93407, USA

**Keywords:** type 2 diabetes mellitus, prediabetes, Mediterranean diet, whey protein, physical activity, obesity, body composition, systemic inflammation

## Abstract

*Background and Objectives*: Type 2 diabetes mellitus (T2DM) is a chronic condition recognized as the inability to maintain glucose homeostasis, typically presenting with insulin resistance and systemic inflammation. With the prevalence of T2DM and major risk factors, such as prediabetes and obesity, increasing each year, the need to address risk factor reduction strategies is crucial. *Materials and Methods*: Twenty-two men and women, overweight-to-obese adults (BMI mean: 26.1–31.6) (age range mean: 44.6–51.8) with T2DM, indicators of prediabetes, or who were metabolically healthy, participated in Cal Poly’s Nutrition and Exercise in Type 2 Diabetes (CPNET) study. There were no significant differences in terms of age, BMI, or sex distribution among the groups at the baseline. This study’s protocol included following a Mediterranean-style diet, the daily consumption of a high-quality whey protein supplement, and physical activity recommendations for 16 weeks. Body composition data, via dual-energy X-ray absorptiometry (DXA), and fasting blood samples were collected at the baseline and following the intervention. Due to restrictions associated with the outbreak of the COVID-19 pandemic, only 13 of the 22 participants who started this study were able to return for the second data collection to complete this study following the 16-week intervention. *Results*: The prediabetic and T2DM groups exhibited reductions in their fasting plasma glucose (12.0 mg/dL reduction in the prediabetic group; 19.6 mg/dL reduction in the T2DM group) to that of normal and prediabetic levels, respectively, while the T2DM group also demonstrated improvement in their hemoglobin A1c (reduced from 6.8% to 6.0%) to prediabetic levels. Additionally, the metabolically healthy, overweight group exhibited significant improvements in adiposity, while the obese prediabetic and T2DM groups showed non-significant improvements in all the measured metrics of body composition. No significant changes were observed in the inflammatory biomarkers (*p*-values ranged from 0.395 to 0.877). *Conclusions*: Collectively, our results suggest that adherence to a well-balanced, nutritious diet and activity may improve the parameters of glycemic control and provide benefits to body composition that help to manage and prevent the development of T2DM. Our study was able to yield significant findings signifying that the effects of a Mediterranean-style diet are observed even for a more conservative sample size.

## 1. Introduction

Type 2 diabetes mellitus (T2DM) is a chronic dysregulation of glucose metabolism, typically due to insulin resistance (IR), affecting millions of individuals worldwide [1,2]. The prevalence of diabetes has steadily increased in the past few decades, with over 536 million recorded cases of diabetes and 541 million recorded cases of prediabetes worldwide in 2021 [2,3]. Long-term uncontrolled hyperglycemia, as seen in sub-optimally managed T2DM, can injure the delicate vasculature of the eyes, kidneys, and nervous system, leading to complications commonly seen in diabetes, such as blurred vision/blindness, chronic kidney disease (CKD) and, in the advanced stages of poorly controlled diabetes, amputations [2]. Damage can also occur to the macrovasculature, promoting the development of cardiovascular disease (CVD), rendering diabetes a cause of CVD and related death secondary to uncontrolled diabetes. These complications cost the United States alone approximately USD 327 billion in 2017 and were mostly attributable to direct medical costs and the loss of productivity in individuals with T2DM.

The risk factors for developing T2DM can be classified into two groups, namely non-modifiable and modifiable [1]. Non-modifiable risk factors are factors typically beyond the control of the individual, including age, sex, genetic predisposition, and ethnicity. Conversely, modifiable risk factors can typically be modulated to a greater or lesser degree through lifestyle and behavioral modifications, such as an improved diet and increased physical activity. A major modifiable risk factor for T2DM is prediabetes, classified as an elevated fasting blood glucose, impaired glucose tolerance, and/or an elevated hemoglobin A1c. Prediabetes typically coexists with other clinical signs of metabolic dysregulation, collectively known as metabolic syndrome (MetSy) [4]. Abdominal obesity, dyslipidemia, hypertension, and elevated fasting plasma glucose are indicators characterizing MetSy. Individuals presenting with three or more of these indicators are typically considered at higher risk for developing T2DM and ensuing CVD. The presence of obesity alone, especially abdominal obesity, has been shown to promote a state of low-grade chronic inflammation and oxidative stress, increasing the risk of T2DM [5]. Obesity status is commonly assessed by calculating the body mass index (BMI), with higher BMIs associated with a greater risk of metabolic diseases, including T2DM and CVD [6]. As the BMI calculation only utilizes height and body weight measurements, the additional measurements of body fat percentage (BF%) and waist circumference are recommended to provide a more accurate assessment of obesity status [7].

Recently, methods have been developed to quantify the specific types of adipose tissue and their location, as these factors can affect the risk for certain diseases. White adipose tissue (WAT), one of the largest, metabolically active endocrine organs in the body, secretes proinflammatory cytokines (i.e., adipokines) to regulate whole-body homeostasis [7]. Under conditions of obesity with excessive WAT stored in the abdominal region (visceral adipose tissue (VAT)), adipocyte hypertrophy leads to a dysregulated WAT metabolism and altered adipokine and cytokine secretion [8]. For example, elevated levels of cytokines interleukin-6 (IL-6), tumor necrosis factor-alpha (TNF-α), and high-sensitivity C-reactive protein (hs-CRP) have been associated with IR and are, therefore, often used as T2DM risk indicators [8].

Currently, there is no cure for diabetes [9]. With the prevalence of T2DM and major risk factors, such as prediabetes and obesity, increasing each year, there is a crucial need to identify strategies for the optimal management and prevention of this condition. Adherence to a healthy dietary pattern and performing regular physical activity may provide a strategy for increasing insulin sensitivity, decreasing adiposity, and decreasing the inflammatory response associated with metabolic dysregulation [6,10]. A Mediterranean-style diet (MSD) is a primarily plant-based diet derived from the populations residing in the regions that border the Mediterranean Sea [10,11,12]. Although MSDs differ within the Mediterranean region, an MSD typically consists of a high intake of vegetables, fruits and nuts, whole grains, legumes, fish, and poultry. Monounsaturated fats from olive oil are the main source of dietary lipids, and alcohol is consumed in low-to-moderate quantities, mostly as red wine. An MSD is typically low in red meat and non-fermented dairy products. The long-term adherence to an MSD has been shown to reduce the risk of T2DM, as well as CVD, various cancers, and all-cause mortality [11]. Recently, there has been growing interest in the possible insulinotropic and appetite-suppressing effects of dietary whey protein for individuals with hyperglycemia and T2DM [13,14]. Whey protein is a good source of all nine essential amino acids and is absorbed quickly and efficiently in the small intestine, making it a high-quality “complete” protein. Whey protein has been shown to promote anabolism and to have rapid effects on glucose metabolism, preventing postprandial hyperglycemia and improving insulin sensitivity with similar efficacy to anti-diabetic medications.

Cal Poly’s Nutrition and Exercise in Type 2 Diabetes (CPNET) study was designed to assess whether certain changes in lifestyle could improve the metabolic status of individuals with T2DM and prediabetes. More specifically, this study was conducted to evaluate the effectiveness of a long-term adherence to an MSD, the daily consumption of a high-quality whey protein supplement, and regular physical activity on the parameters of glycemic control, the metrics of body composition, and the biomarkers of systemic inflammation in obese individuals with T2DM and prediabetes. This CPNET study additionally sought to investigate if improvements in metabolic status, as assessed by using fasting plasma glucose, hemoglobin A1c, and fasting plasma insulin, were associated with improvements in an individual’s body composition metrics and/or inflammatory status. A novelty of our study is the inclusion of prediabetic participants, a currently understudied group in diabetes research.

The primary objective of this CPNET study was to investigate the effects of modifications to diet on the markers of glycemic control, inflammation, and body composition in individuals with T2DM and prediabetes, as well as in healthy individuals. The secondary objectives of this study were to determine which group—the metabolically healthy, prediabetic, or T2DM—demonstrated the most improvement, and if the improvements in the measured outcomes occurred in a progressive manner among the healthy, prediabetic, and T2DM groups, which is pertinent to glycemic control. We hypothesized that a Mediterranean-style diet would improve glycemic control in the participants with prediabetes and T2DM.

## 2. Materials and Methods

Approval by the Institutional Review Board for Human Subjects Research at Cal Poly (Protocol Number: 2018-131) was obtained for all the experimental procedures and written informed consent was obtained from all the participants.

### 2.1. Participants

Individuals living in San Luis Obispo County, California, were recruited to participate in Cal Poly’s Nutrition and Exercise in Type 2 Diabetes (CPNET) study. E-mail advertisements and flyers were distributed to university students, faculty, and staff, and advertisements were published in local newspapers, hospitals, and clinics. Eligible participants were men and women between the ages of 18 and 65 years with either a medical physician’s diagnosis of T2DM, indicators of prediabetes, or who were metabolically healthy (indicated by fasting plasma glucose (FPG), hemoglobin A1c (HbA1c), and fasting plasma insulin (FPI) values within a normal physiological range). Participants were excluded if they were current smokers, had abnormally high blood pressure (systolic > 130 mm Hg, diastolic > 80 mm Hg), had received a diagnosis of a chronic medical condition other than T2DM, or were taking medications used to treat a significant health condition other than T2DM. Prior to beginning this study, each participant completed a health screening questionnaire via telephone with a member of the research team, where they were required to confirm that they had been cleared for exercise by their physician. Any individual who could not confirm medical clearance for exercise was excluded from this study. Following the initial recruitment period, twenty-two adults (12 female, 10 male) were determined eligible to participate and provided written informed consent. Subsequent recruitment periods were canceled due to COVID-19 restrictions beginning. Thus, the dropout rate was not affected by typical reasons, but rather by the unprecedented circumstances due to the COVID-19 pandemic, lock down policies, and an unwillingness to continue and/or return to participation in this study due to fear of the pandemic.

### 2.2. Experimental Protocol

All the data for this CPNET pilot study were collected at two time points: at the baseline and following the 16-week intervention period. Prior to beginning this study, each participant met with a member of the research team and was provided with a personalized nutrition plan based on a Mediterranean-style diet (MSD) to be consumed for 16 weeks. The guidelines for the MSD provided to participants included consuming 4–8 servings per day of non-starchy vegetables, 4–6 servings per day of whole grains and starchy vegetables, 2–3 servings per day of fruits, 2–4 servings per day of nuts and legumes, 1–3 servings per day of low-fat dairy products, 2–3 servings per week of fish or shellfish, 1–3 servings per week of poultry, and 4–6 servings per day of unsaturated fats. Participants were instructed to limit their consumption of saturated fats and added sugars, to reduce their servings of red meat to 4 or fewer per week, and to limit the consumption of alcohol to, at most, one glass of red wine per day. Elizabeth Stewart Hands and Associates (ESHA; version 11.11.32) software was utilized to calculate the daily estimated energy requirement (EER) for each participant. If weight loss was a goal of the participant, the EER was reduced by 500 kcal per day. Additionally, participants were provided with a whey protein powder supplement (supplied by Glanbia Nutritionals) with a shaker cup, and instructed to consume 25 g per day of whey protein powder in 12 ounces of skim milk, almond milk, or water immediately following a bout of exercise. On days when no exercise was performed, the whey protein drink was to be consumed as a morning or afternoon snack.

During the intervention, the participants were encouraged to adhere to the *Physical Activity Guidelines for Americans* [15]. The participants were encouraged to perform aerobic exercise of moderate intensity for 150–300 min per week, or of vigorous intensity for 75–150 min per week, preferably dispersed throughout the weekdays. The participants were instructed to perform 50% of their physical activity as aerobic exercise (e.g., running and cycling), and the remaining 50% as resistance training (e.g., weightlifting and yoga). Examples of light-intensity exercise included: walking, golfing, and stretching. Examples of moderate-intensity exercise included: carrying light loads, cycling at a regular pace, and doubles tennis. Examples of vigorous-intensity exercise included: lifting heavy weights, digging, aerobic exercise, running, and cycling at a fast pace.

### 2.3. Analyses of Parameters of Glycemic Control and Systemic Inflammation

Following a 10–12 h overnight fast, morning blood samples were collected from the eligible participants at Pacific Diagnostic Laboratories in San Luis Obispo, California. Blood samples were drawn using venipuncture in the antecubital fossa by a licensed phlebotomist and analyzed for fasting plasma levels of glucose and insulin, fasting serum levels of HbA1c, high-sensitivity C-reactive protein (hs-CRP), interleukin-6 (IL-6), and tumor necrosis factor-alpha (TNF-α). Due to technical issues, the laboratory was unable to calculate the fasting plasma insulin (FPI) and TNF-α values for one female participant in the control group at the baseline, and the TNF-α value for one male participant in the prediabetic group post-intervention.

Participants were assigned to one of three groups based on their FPG and HbA1c values following the National Institute of Diabetes and Digestive and Kidney Diseases (NIDDK) guidelines for the classification of diabetes based on glycemic control [16] (see Supplementary Table S1): (1) the metabolically healthy group (control; *n* = 10); (2) the prediabetes group (preDM; *n* = 5); or (3) the type 2 diabetes mellitus group (T2DM; *n* = 7). Participants were characterized at the baseline based on their age, sex, and body mass index (BMI).

### 2.4. Body Composition and Anthropometric Analyses

Following blood collection, body composition was analyzed via a complete body scan using dual-energy X-ray absorptiometry (DXA) at Cal Poly’s Food Science and Nutrition Department’s Nutrition and Health Assessment Laboratory. All female participants were required to take a pregnancy test upon arrival to ensure negative pregnancy results prior to the scan. Complete body scans were obtained with the Lunar iDXA system (General Electric Company). All DXA scans were performed by the same licensed technician following phantom spine calibration to ensure the optimal performance of the instrument. Data collected from the DXA scans utilized in this analysis include body fat percent region (BF%) and visceral adipose tissue mass (VAT, pounds (lbs)).

The anthropometrics for height (inches) and body weight (lbs) were measured with a beam scale and a stadiometer, and waist circumference (WC, inches) was measured using a standard, non-stretch measuring tape. Body mass index (BMI) was calculated using the formula presented below:

BMI = [Weight(lbs) × 703]/[height(inches)]^2^

### 2.5. Statistical Analyses 

To detect differences between the control, preDM, and T2DM groups at the baseline and following the 16-week intervention, for all of the following parameters—FPG, HbA1c, FPI, hs-CRP, IL-6, TNF-α, body weight, WC, BMI, BF%, and VAT—we used one-way between-groups analysis of variance (ANOVA) in SPSS 27 (IBM Corp., Armonk, NY, USA). Post hoc comparisons using Tukey’s HSD test were performed to correct for multiple comparisons and to identify where significant differences between the groups occurred. To detect changes from the baseline to week 16 within each group (control, preDM, and T2DM), the following parameters, including FPG, HbA1c, FPI, hs-CRP, IL-6, TNF-α, body weight, WC, BMI, BF%, and VAT, were analyzed using paired-samples *t*-tests in SPSS 27 (IBM Corp.). All data are presented as the mean ± standard deviation (SD) and results with a *p*-value of less than or equal to 0.05 were considered significant.

## 3. Results

**Figure 1:**
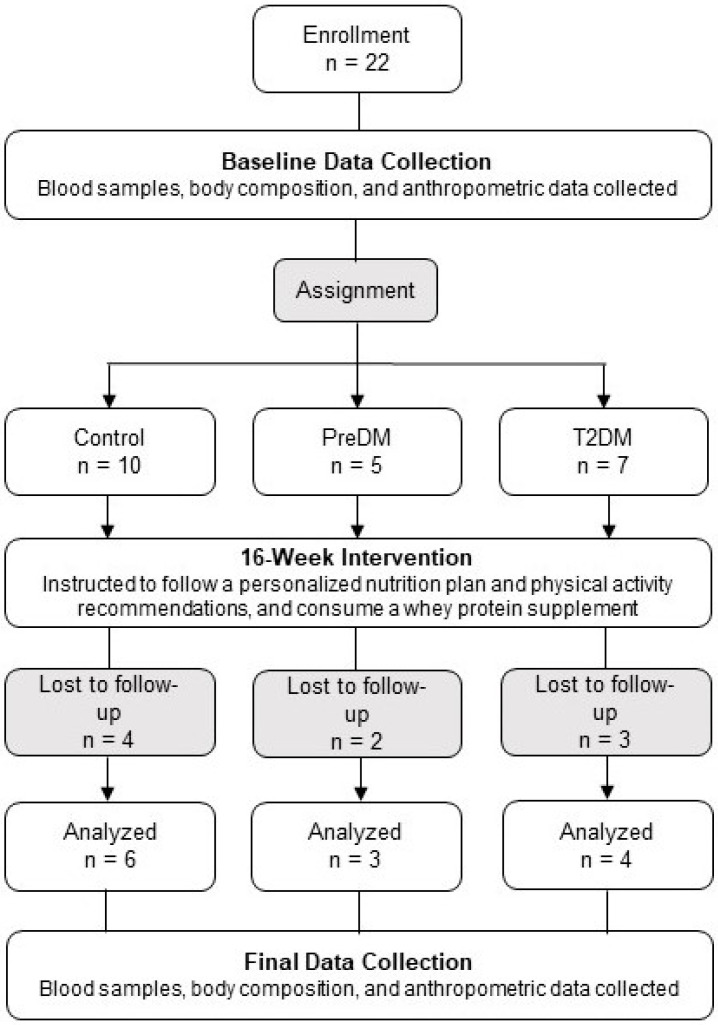
Flowchart of this CPNET study, which includes participants enrolled at baseline (*n* = 22) and analyzed following the 16-week intervention (*n* = 13). The personalized nutrition plans were based on a Mediterranean-style diet (MSD). Physical activity recommendations were based on the *Physical Activity Guidelines for Americans* [15].

Twenty-two individuals (12 females, 10 males; distributed as follows into three groups: control, *n* = 10; preDM, *n* = 5; T2DM, *n* = 7) began the 16-week intervention at staggered time points between March 2019 and March 2020, and were characterized at the baseline based on their age, sex, and BMI (Figure 1; Table 1). There were no statistically significant differences in the distribution of these characteristics (sex, age, and BMI) among the three groups (Table 1), thus, making them unlikely to constitute confounding factors. Recruitment for this study was curtailed by COVID-19 public health-related state mandates, thus, significantly reducing the number of individuals able to participate in and complete this study. Nine individuals (four from the control group, two from the preDM group, and three from the T2DM group) discontinued their participation due to a variety of reasons, including COVID-19 restrictions, lockdown policies, and fear of participating due to the risk of COVID-19. Thirteen participants (seven females, six males; control, *n* = 6; preDM, *n* = 3; T2DM, *n* = 4) completed the 16-week intervention and returned for the final data collection.

### 3.1. Parameters of Glycemic Control

#### 3.1.1. Fasting Plasma Glucose

At the baseline, significant differences in the fasting plasma glucose (FPG) were observed between the control and preDM groups (*p* = 0.047), and between the control and T2DM groups (*p* < 0.0001; Table 2). These data confirm the allocation of participants into the appropriate groups at the baseline. Following the 16-week intervention, the FPG was reduced in the preDM and T2DM groups to the point where no significant differences persisted between any of the groups (*p* > 0.05). The decrease in the FPG to normal levels observed within the preDM group is indicative of a trend with a large effect size (pre: 106.0 ± 9.11 mg/dL, post: 94.0 ± 7.55 mg/dL; *p* = 0.065, Cohen’s d = 2.144), and the T2DM group showed a non-significant decrease in mean FPG to prediabetic levels (pre: 126.1 ± 26.98 mg/dL, post: 106.5 ± 31.17 mg/dL; *p* = 0.232). The results suggest that the long-term adherence to the combined intervention tended to positively affect blood glucose regulation in those with preDM and T2DM.

#### 3.1.2. Hemoglobin A1c

Significant differences in the hemoglobin A1c (HbA1c) were observed between the control and T2DM groups (*p* = 0.001), and between the preDM and T2DM groups at the baseline (*p* = 0.044; Table 2), confirming the initial allocation of participants into the appropriate groups. Following the 16-week intervention, significant differences in the HbA1c between the control and T2DM groups persisted, but were relatively less significant than at the baseline (*p* = 0.031); the mean HbA1c of the T2DM group had decreased to the point where there was no longer a significant difference between the preDM and T2DM groups post-intervention (*p* > 0.05). Although the decrease in the mean HbA1c within the T2DM group was non-significant (*p* = 0.391), it did decrease from the diabetic to prediabetic level (pre: 6.8 ± 1.20%, post: 6.0 ± 0.75%). Thus, the long-term adherence to the combined intervention may reduce the severity of glycation phenomena associated with T2DM.

#### 3.1.3. Fasting Plasma Insulin

There were no significant differences in the mean fasting plasma insulin (FPI) levels observed between any of the groups at the baseline; however, the T2DM group tended to have a higher FPI compared to the control (*p* = 0.093; Table 2). This trending difference between the T2DM and control groups failed to persist at the week 16 follow-up, due to a non-significant decrease in the mean FPI within the T2DM group. Additionally, non-significant decreases in the FPI were observed within all three groups from the baseline to week 16 (*p* > 0.05). These results lend further support to the hypothesis that the combined intervention may benefit glucose regulation through improvements in insulin sensitivity.

### 3.2. Systemic Inflammation

At the baseline and the week 16 follow-up, there were no significant differences found between any of the groups for the high-sensitivity C-reactive protein (hs-CRP), interleukin-6 (IL-6), or tumor necrosis factor-alpha (TNF-α; Table 3). However, improvements in hs-CRP within the preDM group did suggest a possible benefit to the intervention, as the mean hs-CRP was reduced from levels indicating a high risk for cardiovascular (CV) complications to an average risk (pre: 3.6 ± 3.62 mg/L, post: 2.2 ± 3.02 mg/L; *p* = 0.414). Although decreases in the mean hs-CRP were observed within the control and T2DM groups as well, the changes were non-significant and did not indicate an improvement in risk for CV complications. Thus, the combined intervention was not able to significantly reduce the inflammatory biomarkers within the time parameters of this study. This is an indication that potentially significant improvements in inflammatory status would require longer exposure to such an intervention; given the dynamic nature of inflammatory responses, this is biologically plausible.

### 3.3. Body Composition and Anthropometrics

At the baseline and at the week 16 follow-up, there were no significant differences found among any of the groups for body weight, waist circumference (WC), body mass index (BMI), body fat percent region (BF%), or visceral adipose tissue mass (VAT; Table 4). However, significant improvements were observed within the control group for several metrics of body composition. Following the intervention, the mean body weight of the control group was significantly reduced from 191.7 ± 49.15 lbs to 167.1 ± 43.28 lbs, with a large effect size (*p* = 0.030, Cohen’s d = 1.221); the mean BF% was significantly reduced from 36.6 ± 6.46% to 34.6 ± 8.00%, with a large effect size (*p* = 0.003, Cohen’s d = 2.204). Additionally, the control group demonstrated a significant decrease in mean BMI from 28.6 ± 5.86 to 26.2 ± 5.90, with a large effect size (*p* = 0.043, Cohen’s d = 1.104); however, their classification based on their BMI remained in the overweight category. The results provide evidence that the long-term adherence to the combined intervention reduces adiposity in metabolically healthy, overweight adults, possibly preventing the development of metabolic dysfunction associated with T2DM.

In the preDM and T2DM groups, no significant changes in body weight, WC, BMI, BF%, or VAT were observed; however, slight improvements were found for each metric. In both groups, non-significant decreases were observed in the mean body weight, BF%, and VAT. Both groups also demonstrated non-significant decreases in mean BMI, from the obese class I category to the overweight category (preDM—pre: 30.7 ± 6.46, post: 28.4 ± 5.19, *p* = 0.592; T2DM—pre: 31.6 ± 5.30, post: 28.6 ± 5.48, *p* = 0.136). Additionally, the T2DM group demonstrated a trend of decreased mean WC, from 42.7 ± 6.45 inches to 37.4 ± 5.31 inches, with a large effect size (*p* = 0.058, Cohen’s d = 1.500).

## 4. Discussion

Cal Poly’s Nutrition and Exercise in Type 2 Diabetes (CPNET) study investigated the effects of lifestyle modification (diet and physical activity) on body composition, and the regulation of glucose metabolism and systemic inflammation in overweight-to-obese healthy, prediabetic, and type 2 diabetic individuals. 

### 4.1. Glycemic Control

Similar studies have been conducted to investigate the effectiveness of lifestyle modifications for the management and prevention of T2DM. The improvements to glycemic control observed in our study are consistent with the findings of a systematic review and meta-analysis of studies investigating the effects of adherence to a Mediterranean diet (MD), as compared to mainly low-fat control diets, conducted by Esposito et al. in 2015 [17]. The meta-analysis found that an MD was effective at improving HbA1c and cardiovascular disease (CVD) risk factors in participants with T2DM, although the authors noted the low availability of long-term randomized controlled trials (RCTs). Similarly, Huo et al. (2015) performed a meta-analysis of nine RCTs assessing the impact of a Mediterranean-style diet (MSD) versus a control diet on glycemic control and body composition in participants with T2DM; they concluded that an MSD is effective at reducing fasting glucose and insulin levels, HbA1c, body weight, and BMI, with additional improvements to lipid profiles [18]. However, this meta-analysis was limited by variations in the control diets of the included studies, and a low number of trials for some of the outcome variables. 

A network meta-analysis of eight RCTs by Carter et al. (2014) also found that an MD was beneficial for reducing HbA1c in individuals with T2DM or who were at high risk for T2DM and/or CVD, compared to usual care, but it was not more effective than a Paleolithic diet [19]. However, the researchers ultimately determined that the results were inconclusive, as the publication bias was not assessed due to the low number of available studies, narrow confidence intervals attributable to the method of network meta-analysis, and because several of the studies encouraged exercise and stress management in combination with an MD, making it difficult to attribute the beneficial results to the dietary changes alone. A meta-analysis of 16 RCTs by Esposito et al. (2011), which investigated the effects of an MD on the metrics of body composition, found that an MD was effective at significantly reducing body weight and BMI, especially when followed long-term, or when combined with energy restriction or exercise [20]. The high variability in the health status of the participants, however, limited the specificity of these findings. It is important to note that all the meta-analyses discussed herein mention a lack of homogeneity between the MD or MSD interventions utilized in the studies selected, although all the intervention diets did present the basic characteristics of a standard MD. It is important to note, however, that the way the MD is perceived in its originally derived regions (Greece and southern Italy) is more of a lifestyle that, in addition to its dietary patterns, also includes physical activity, a slow pace, afternoon rest, and an attitude towards time that arguably renders less stress, and promotes socialization with friends and family, thus, generating a solid support group [21].

A few observational studies have demonstrated a relationship between the consumption of an MD or MSD and improvements in glucose handling and risk factors for chronic diseases. A cross-sectional case–control study conducted by Murray et al. (2013) found that the diet of T2DM cases resembled that of a “Western-style” diet, while the non-T2DM controls had significantly higher Mediterranean diet (MD) scores and a better overall quality of diet [22]. The study was limited by using 3-day food records, which can lead to the misreporting of dietary intake, a small sample size, significant differences in the BMI between the groups, and an unbalanced ratio of men to women between the cases and controls. Another observational study by Vitale et al. (2018) analyzed the baseline data from the Thiazolidinediones or Sulphonylureas and Cardiovascular Accidents Intervention Trial (TOSC.IT) in Italy [23]. The study found an association between a high relative Mediterranean diet score (rMED) and improved glucose control, lower BMI, and lower prevalence of CVD risk factors in individuals with T2DM. Interestingly, the study found that the observed benefits corresponded to the MD pattern as a whole, and not to the individual nutrients contained in the MD. A systematic review and meta-analysis of one RCT, nine prospective studies, and seven cross-sectional studies performed by Koloverou et al. (2014) evaluated the effects of an MD, specifically, on the incidence of T2DM, and concluded that the MD was effective at reducing T2DM incidence by 23% [24]. However, the wide variation in the health status of the study participants, from apparently healthy individuals to individuals at high risk for T2DM and/or CVD, contributed to the high heterogeneity among the studies considered, and the inclusion of only one RCT limits the strength of these findings.

#### 4.1.1. Strengths—Considerations

A major strength of this CPNET study is the inclusion of individuals with prediabetes, an understudied population vital for developing strategies to combat the rise of T2DM diagnoses worldwide [25]. Most studies conducted on diabetes thus far have included only T2DM populations; however, a few large studies targeting lifestyle-related factors, such as diet and obesity, including the Diabetes Prevention Program (DPP), have demonstrated the effective prevention of T2DM in prediabetic populations. The inclusion of healthy, prediabetic, and T2DM groups in this CPNET study allowed us to observe which groups specifically responded to the combined intervention, and whether there was a progressive improvement in this study’s outcomes depending on the initial metabolic status. Our results indicate that the combined intervention had the most significant effect on body composition in the metabolically healthy, overweight group. However, non-significant improvements in body composition did correspond with improvements to the parameters of glycemic control in the prediabetic and T2DM groups. We also observed a progressive improvement in glycemic control, as indicated by the reduction in fasting plasma glucose (FPG) and hemoglobin A1c (HbA1c) in the T2DM group compared to that of the prediabetic group, while the FPG in the prediabetic group decreased to normal levels following the intervention. These results support the inclusion of prediabetic individuals in T2DM research and suggest that improvements to lifestyle factors may provide a potential point of intervention to prevent T2DM in at-risk populations. Between 2013 and 2016, 34.5% of the adult population of the United States was estimated to have prediabetes [26]; therefore, focusing on prophylactic measures in the prediabetic population may provide a strategy for slowing the rate of T2DM incidence. 

Other strengths of this CPNET study include the addition of a high-quality whey protein supplement in the dietary intervention, to enhance muscle metabolism following exercise, and the combination of long-term dietary and physical activity recommendations in the intervention protocol. Moreover, we were able to utilize well-established recommendations for improving multiple metrics of health in both healthy individuals and those diagnosed with chronic diseases by including the *Physical Activity Guidelines for Americans* in this CPNET study [15].

#### 4.1.2. Limitations—Considerations

This CPNET study was limited by the small sample sizes of the participant groups. This was mostly due to the constraints imposed by the global COVID-19 pandemic, which limited recruitment, led to participant dropout during the intervention period, and resulted in the early termination of recruitment for this study. Paired-samples *t*-tests were utilized to detect changes from the baseline to week 16 within each group; consequently, only the participants who completed both the pre- and post-assessments were able to be included in these analyses, reducing the sample size even further. Therefore, due to the small sample sizes of the groups in our study, we are unable to conclude deterministic relationships between the combined treatment and outcome variables; rather, our data are indicative of significant associations only.

Although 3-day food records were collected and analyzed for their nutritional content at the baseline, the food records collected at week 16 were not analyzed. However, there are recent studies that have demonstrated a positive association between adherence to a Mediterranean diet and a lower risk for T2DM [27,28]. In addition, the participants were recommended to follow the *Physical Activity Guidelines for Americans* [15] during the intervention period, and an assessment of their physical activity was performed at the baseline and following the intervention; however, no analyses of these results were performed, making it difficult to evaluate the participants’ actual physical activity status during the intervention. 

Other limitations include no stratification of the data by sex, as there are typically differences in lean body mass, body fat percentage, and waist circumference between men and women, and the lack of data relating to the duration of diabetes diagnosis. Additionally, markers for skeletal muscle metabolism (e.g., mitochondrial metabolites) were not measured, as muscle biopsies were not obtained during this study. Lastly, utilizing high-sensitivity C-reactive protein (hs-CRP) as a marker of inflammation has its own constraints, as hs-CRP is an acute-phase protein that can have high within-person variability, thus requiring measurements at two separate time points to adequately reflect an individual’s hs-CRP status [29]. 

#### 4.1.3. Future Directions

This CPNET study consisted of a preliminary pilot study demonstrating the potential of lifestyle modifications, specifically an MSD and increased physical activity, to manage and prevent T2DM. Future studies should focus on the recruitment of participants with T2DM and prediabetes, as well as metabolically healthy individuals as the control. Additionally, the inclusion of overweight and obese participants is crucial to explore how changes in the various metrics of body composition affect glycemic control and levels of systemic inflammation. There is also a need to elucidate whether initially high levels of systemic inflammation in obese individuals with T2DM and prediabetes can be lessened by alterations to diet and physical activity, and if this corresponds to improvements in glycemic control and insulin sensitivity. Moreover, it would be interesting to investigate how a changing microbiome may influence the progression towards diabetes, and how strong such a relationship is, [30] as well as how protein and specific amino acids may influence body composition and insulin resistance progression for the improvement of prediabetic outcomes [31].

## 5. Conclusions

This 16-week CPNET pilot study demonstrates the potential of a Mediterranean-style dietary pattern, supplemented with whey protein and combined with regular physical activity, to positively affect the metrics of body composition, blood glucose regulation, and insulin sensitivity in obese individuals with prediabetes and type 2 diabetes mellitus (T2DM). The combined intervention was effective at decreasing the markers of glycemic control in the prediabetic and T2DM participants, while having the most significant impact on body composition and adiposity in the metabolically healthy, overweight participants. Additionally, non-significant improvements to all the utilized measures of body composition were observed in the T2DM and prediabetic participants. These improvements may be beneficial for preventing and managing T2DM and, thus, preventing the complications associated with T2DM. Our findings lend support to the use of diet- and lifestyle-modification recommendations by healthcare providers as a potential first line of defense against, and as a treatment for, T2DM.

## Figures and Tables

**Table 1:** Characteristics of the study participants at baseline.

	Group			
	Control *n* = 10	PreDM *n* = 5	T2DM *n* = 7	*p*-Value
Age (years) ^†^	44.6 ± 10.3	51.8 ± 11.5	46.9 ± 14.8	0.565
Sex (% female)	60	40	57.1	0.781
Body Mass Index (BMI) ^†^	28.6 ± 5.86	30.7 ± 6.46	31.6 ± 5.30	0.569

^†^ Data presented as mean ± standard deviation (SD) obtained from one-way between-groups ANOVA with post-hoc Tukey’s HSD test.

**Table 2:** Parameters of glycemic control at baseline and the week 16 follow-up ^†^.

		Group		
		Control	PreDM	T2DM
Fasting plasma glucose (FPG; mg/dL)	Baseline:	82.6 ± 7.66 ^a^	106.0 ± 9.11 ^b^	126.1 ± 26.98 ^b^
	Week 16:	81.7 ± 5.72	94.0 ± 7.55	106.5 ± 31.17
	*p*-value ^1^:	0.790	0.065	0.232
Hemoglobin A1c (HbA1c; %)	Baseline:	5.2 ± 0.29 ^a^	5.7 ± 0.30 ^a^	6.8 ± 1.20 ^b^
	Week 16:	5.1 ± 0.34 ^A^	5.7 ± 0.06 ^A,B^	6.0 ± 0.75 ^B^
	*p*-value ^1^:	0.224	1.000	0.391
Fasting Plasma Insulin (FPI; mU/L)	Baseline:	8.4 ± 4.35	10.7 ± 5.86	17.5 ± 12.27 ^$^
	Week 16:	5.4 ± 2.93	7.0 ± 4.59	10.8 ± 7.25
	*p*-value ^1^:	0.162	0.209	0.216

^†^ Data presented as mean ± standard deviation (SD). Different lowercase letters indicate a significant difference of *p* ≤ 0.05 between groups at baseline (*n* = 22) and different uppercase letters indicate a significant difference of *p* ≤ 0.05 between groups at week 16 (*n* = 13), obtained from one-way between-groups ANOVA with post-hoc Tukey’s HSD test. ^$^ *p* < 0.1 compared with control group at baseline (*n* = 22). ^1^ Within-group comparison of mean values at baseline versus week 16, obtained from paired-samples *t*-test.

**Table 3:** Comparisons of inflammatory biomarkers at baseline and the week 16 follow-up ^†^.

		Group		
		Control	PreDM	T2DM
High-sensitivity C-reactive protein (hs-CRP; mg/L)	Baseline:	2.4 ± 2.10	3.6 ± 3.62	5.7 ± 6.30
	Week 16:	1.5 ± 0.84	2.2 ± 3.02	4.0 ± 3.70
	*p*-value ^1^:	0.408	0.414	0.862
Interleukin-6 (IL-6; pg/mL)	Baseline:	1.8 ± 2.47	2.6 ± 1.74	3.8 ± 3.85
	Week 16:	1.2 ± 0.50	2.0 ± 1.39	4.1 ± 4.21
	*p*-value ^1^:	0.814	0.877	0.718
Tumor necrosis factor-alpha (TNF-α; pg/mL)	Baseline:	1.0 ± 0.33	1.3 ± 0.19	1.4 ± 0.51
	Week 16:	1.2 ± 0.20	1.1 ± 0.28	1.3 ± 0.39
	*p*-value ^1^:	0.749	0.395	0.492

^†^ Data presented as mean ± standard deviation (SD). ^1^ Within-group comparison of mean values at baseline versus week 16, obtained from paired-samples *t*-test.

**Table 4:** Comparisons of anthropometrics and body composition at baseline and the week 16 follow-up ^†^.

		Group		
		Control	PreDM	T2DM
Body Weight (lbs)	Baseline:	191.7 ± 49.15	206.4 ± 41.31	198.9 ± 47.54
	Week 16:	167.1 ± 43.28	196.5 ± 40.82	171.8 ± 49.92
	*p*-value ^1^:	0.030	0.603	0.135
Waist Circumference (WC; inches)	Baseline:	39.8 ± 6.28	42.0 ± 7.89	42.7 ± 6.45
	Week 16:	37.2 ± 6.62	38.8 ± 8.01	37.4 ± 5.31
	*p*-value ^1^:	0.239	0.431	0.058
Body Mass Index (BMI)	Baseline:	28.6 ± 5.86	30.7 ± 6.46	31.6 ± 5.30
	Week 16:	26.2 ± 5.90	28.4 ± 5.19	28.6 ± 5.48
	*p*-value ^1^:	0.043	0.592	0.136
Body Fat (percent region, BF%)	Baseline:	36.6 ± 6.46	34.7 ± 8.40	38.4 ± 4.93
	Week 16:	34.6 ± 8.00	30.6 ± 7.73	36.3 ± 5.78
	*p*-value ^1^:	0.003	0.986	0.199
Visceral Adipose Tissue (VAT; mass, lbs)	Baseline:	2.5 ± 1.54	3.73 ± 2.49	4.35 ± 2.24
	Week 16:	1.83 ± 1.24	2.82 ± 2.92	2.99 ± 2.14
	*p*-value ^1^:	0.127	0.148	0.188

^†^ Data presented as mean ± standard deviation (SD). ^1^ Within-group comparison of mean values at baseline versus week 16, obtained from paired-samples *t*-test.

## Data Availability

Not applicable.

## Supplementary Materials

The following supporting information can be downloaded at: https://www.mdpi.com/article/10.3390/medicina59101882/s1, Table S1: NIDDK guidelines for the classification of type 2 diabetes mellitus and prediabetes.

## Abbreviations

Type 2 diabetes mellitus (T2DM), insulin resistance (IR), chronic kidney disease (CKD), cardiovascular disease (CVD), metabolic syndrome (MetSy), body mass index (BMI), fat percentage (BF%), white adipose tissue (WAT), visceral adipose tissue (VAT), interleukin-6 (IL-6), tumor necrosis factor-alpha (TNF-α), high-sensitivity C-reaction protein (hs-CRP), Mediterranean-style diet (MSD), American Heart Association (AHA), American Diabetes Association (ADA), Cal Poly’s Nutrition and Exercise in Type 2 Diabetes (CPNET), California Polytechnic State University in San Luis Obispo (Cal Poly), fasting plasma glucose (FPG), hemoglobin A1c (HbA1c), fasting plasma insulin (FPI), Elizabeth Stewart Hands and Associates (ESHA), estimated energy requirement (EER), International Physical Activity Questionnaire (IPAQ), National Institute of Diabetes and Digestive and Kidney Diseases (NIDDK), dual-energy X-ray absorptiometry (DXA), United States Department of Agriculture (USDA), analysis of variance (ANOVA), standard deviation (SD), waist circumference (WC), randomized controlled trials (RCTs), Mediterranean diet (MD), relative Mediterranean diet score (rMED), non-insulin-dependent diabetes mellitus (NIDDM), homeostasis model assessment-estimated insulin resistance (HOMA-IR).
