# Supplementary material for: A Mediterranean-Style Diet Improves the Parameters for the Management and Prevention of Type 2 Diabetes Mellitus
Source: Medicina (Kaunas). 2023 Oct 23;59(10):1882. doi: 10.3390/medicina59101882 (PMC10608307; doi:10.3390/medicina59101882)
Supplement: Supplementary file 1 [file medicina-59-01882-s001.zip › medicina-2667726-supplementary.pdf]

Supplemental Materials

Table S1. NIDDK guidelines for the classification of type 2 diabetes mellitus and prediabetes.

Diagnosis	Hemoglobin A1c (HbA1c, %)	Fasting Plasma Glucose (FPG, mg/dL)[a]	Oral Glucose Tolerance Test (OGTT, mg/dL)[b]	Random Plasma Glucose test (RPG, mg/dL)[c]
Normal	< 5.7	≤ 99	≤ 139	-
Prediabetes	5.7 - 6.4	100 - 125	140 - 199	-
Diabetes	≥ 6.5	≥ 126	≥ 200	≥ 200

[a]Performed following a fast of at least 8 hours. [b]2-hour plasma glucose measured following a bolus of glucose equal to 75 g anhydrous glucose dissolved in water. [c]Performed in patients experiencing hyperglycemic crisis or who present with classic symptoms of hyperglycemia. NIDDK, National Institute of Diabetes and Digestive and Kidney Diseases. Source: NIDDK, 2022.
